# Hypothalamic S-Nitrosylation Contributes to the Counter-Regulatory Response Impairment following Recurrent Hypoglycemia

**DOI:** 10.1371/journal.pone.0068709

**Published:** 2013-07-19

**Authors:** Xavier Fioramonti, Adam Deak, Srinidhi Deshpande, Lionel Carneiro, Chunxue Zhou, Nazish Sayed, Branly Orban, Joshua R. Berlin, Luc Pénicaud, Corinne Leloup, Annie Beuve, Vanessa H. Routh

**Affiliations:** 1 Department of Pharmacology and Physiology, New Jersey Medical School, Newark, New Jersey, United States of America; 2 Centre for Taste and Feeding Behavior (CSGA), UMR 6265 CNRS, 1324 INRA-Université de Bourgogne, Dijon, France; Goethe University, Germany

## Abstract

**Aims:**

Hypoglycemia is a severe side effect of intensive insulin therapy. Recurrent hypoglycemia (RH) impairs the counter-regulatory response (CRR) which restores euglycemia. During hypoglycemia, ventromedial hypothalamus (VMH) production of nitric oxide (NO) and activation of its receptor soluble guanylyl cyclase (sGC) are critical for the CRR. Hypoglycemia also increases brain reactive oxygen species (ROS) production. NO production in the presence of ROS causes protein S-nitrosylation. S-nitrosylation of sGC impairs its function and induces desensitization to NO. We hypothesized that during hypoglycemia, the interaction between NO and ROS increases VMH sGC S-nitrosylation levels and impairs the CRR to subsequent episodes of hypoglycemia. VMH ROS production and S-nitrosylation were quantified following three consecutive daily episodes of insulin-hypoglycemia (RH model). The CRR was evaluated in rats in response to acute insulin-induced hypoglycemia or via hypoglycemic-hyperinsulinemic clamps. Pretreatment with the anti-oxidant N-acetyl-cysteine (NAC) was used to prevent increased VMH S-nitrosylation.

**Results:**

Acute insulin-hypoglycemia increased VMH ROS levels by 49±6.3%. RH increased VMH sGC S-nitrosylation. Increasing VMH S-nitrosylation with intracerebroventricular injection of the nitrosylating agent S-nitroso-L-cysteine (CSNO) was associated with decreased glucagon secretion during hypoglycemic clamp. Finally, in RH rats pre-treated with NAC (0.5% in drinking water for 9 days) hypoglycemia-induced VMH ROS production was prevented and glucagon and epinephrine production was not blunted in response to subsequent insulin-hypoglycemia.

**Conclusion:**

These data suggest that NAC may be clinically useful in preventing impaired CRR in patients undergoing intensive-insulin therapy.

## Introduction

Intensive insulin therapy is used clinically to obtain satisfactory glycemic control in patients with both Type 1 (T1DM) and advanced Type 2 diabetes mellitus (T2DM) in order to avoid long-term complications of hyperglycemia. However, intensive insulin therapy is limited by the induction of iatrogenic hypoglycemia. Hypoglycemia is a profound threat to the brain since glucose is its preferred fuel. In people without diabetes, a fall in blood glucose level is detected both centrally and peripherally and triggers appropriate protective mechanisms including the release of hormones (e.g. glucagon, epinephrine) which stimulate endogenous glucose production and reduce glucose tissue uptake. These mechanisms known as the counter-regulatory response (CRR) prevent and limit hypoglycemia and restore blood glucose level to its physiological set point [1]. Unfortunately, in most patients with diabetes treated with intensive insulin therapy, the CRR is impaired as a consequence of recurrent episodes of hypoglycemia (RH). This impairment is known as hypoglycemia-associated autonomic failure (HAAF). During HAAF, the glycemic threshold for CRR initiation shifts to lower glucose levels. Thus, glucose levels are allowed to drop, without detection, to dangerously low or lethal levels [1], [2], [3]. Although HAAF is the major limiting factor in intensive insulin therapy, the cellular mechanisms involved in its development remain unclear.

In the brain, the ventromedial hypothalamus (VMH) plays a critical role in the initiation of the CRR [4], [5], [6]. The VMH contains specialized neurons which detect changes in extracellular glucose level known as glucose sensing neurons (GSNs) [7], [8], [9], [10]. Among the GSNs, we and others have suggested that glucose-inhibited (GI) neurons, which are excited as glucose level falls, play a major role in the CRR [7]. That is, we showed that the response of VMH GI neurons to decreased glucose was impaired in conditions where the CRR is also impaired such as after RH [11]. In addition, our laboratory showed that the gaseous messenger, nitric oxide (NO) is required for both glucose sensing by GI neurons and full initiation of the CRR [12], [13]. Decreased glucose activates AMP-activated protein kinase (AMPK) in GI neurons leading to phosphorylation of neuronal nitric oxide synthase (nNOS) and NO production. NO then binds to its cytosolic receptor, soluble guanylyl cyclase (sGC) and increases the levels of cyclic guanosine monophosphate (cGMP). Increased cGMP levels are essential for full AMPK activation, chloride channel closure and increased activity of GI neurons [13]. Disruption of any part of the nNOS-NO-sGC signaling pathway impairs glucose sensing by GI neurons and reduces the CRR [12], [13], [14]. In fact, mice lacking nNOS have an impaired CRR and a complete absence of GI neurons [12]. These data are consistent with a key role for NO signaling in GI neurons in the initiation of the CRR.

While sGC mediates many of NO's physiological effects including the regulation of the CRR and glucose sensing in GI neurons, NO also affects other signaling pathways through S-nitrosylation. S-nitrosylation is a post-translational modification consisting of the addition of a NO moiety to a free-thiol cysteine in proteins [15]. S-nitrosylation alters the activity of proteins/enzymes involved in glucose sensing. For instance, S-nitrosylation of the sGC decreases its activity and affinity for NO [16]. S-nitrosylation can occur in the presence of elevated levels of reactive oxygen species (ROS) [15]. Insulin-induced hypoglycemia has been suggested to increase hypothalamic ROS levels and decrease anti-oxidant defenses in normal and diabetic rats [17]. Thus, concomitant VMH NO and ROS production during insulin-induced hypoglycemia may increase S-nitrosylation levels. In this study, we hypothesize that VMH S-nitrosylation of proteins involved in glucose sensing such as sGC contributes to the mechanism(s) underlying the development of HAAF.

## Materials and Methods

### Animals

All procedures were approved by the Institutional Animal Care and Use Committee at the University of Medicine and Dentistry of New Jersey (Newark, NJ, USA) and the University of Burgundy (Dijon, France; ROS measurement only). Adult male Sprague-Dawley rats (100–150 g for *in vivo* experiments; 4–5 weeks old for *in vitro* imaging experiment) were purchased from Charles Rivers. Animals were housed individually and maintained on a 12–12 hour light–dark schedule at 22–23°C with *ad libitum* access to standard chow and water.

### RH protocol

Rats were assigned to four treatment groups as represented in Table 1. Rats were either injected daily subcutaneously with saline or insulin (4 U/kg; regular human; Lilly) for three consecutive days. Using this established RH protocol we have already shown that impaired CRR is associated with impaired sensitivity of VMH GSNs to decreased glucose [11]. The fourth day, VMH ROS production, S-nitrosylation or the CRR were analyzed in response to an acute subcutaneous injection of saline (groups S^3^S and I^3^S) or insulin (4 U/kg; groups S^3^I and I^3^I). Every day of the RH protocol, food was removed for three hours from one hour before the subcutaneous injection to two hours after.

**Table 1:** Representation of different treatment groups used.

		Treatment Days			
		Day 1	Day 2	Day 3	Day 4
**Groups **	**S^3^ S**	Saline	Saline	Saline	Saline
	**S^3^ I**	Saline	Saline	Saline	**Insulin**
	**I^3^ S**	**Insulin**	**Insulin**	**Insulin**	Saline
	**I^3^ I**	**Insulin**	**Insulin**	**Insulin**	**Insulin**

Rats were injected daily with either saline or insulin (4 U/kg; subcutaneously) for four consecutive days.

### N-acetyl-cysteine (NAC) treatment

NAC (Sigma) was prepared fresh daily and given in drinking water (5 g/l) for 9 days prior and during the RH protocol. When normalized to the amount of NAC at 5 g/l consumed per day, this dose of NAC is equivalent to 536±17 mg/day/kg of body weight. This dose of NAC was chosen because it is equivalent to what it is given in human medicine for treatment against paracetamol intoxication per day [18]. It is noteworthy that NAC given at 5 g/l in drinking water decreased fluid intake (water: 136±2.7 *vs* NAC: 107±3.4 ml/day/kg of body weight; n = 4; p<0.05). However, even though NAC decreased water intake, NAC treatment did not alter body weight gain during the treatment (body weight gain: water treated rats: + 24.8±0.9 *vs* NAC treated rats: + 23.5±1.2 g). This is consistent with other studies showing that given at a higher dose does not affect body weight gain [19].

### VMH ROS level measurement

VMH ROS levels were measured as previously described [20]. Briefly, the fourth day of the RH protocol, rats were injected with saline (S^3^S or I^3^S) or insulin (S^3^I or I^3^I; 4 U/kg, subcutaneously) and sacrificed 45 minutes later by decapitation. Brains were quickly removed and the VMH harvested, snap frozen and stored at −80°C. Tissue treatment for ROS determination was performed according to Szabados *et al.* [21]. VMH chunks were incubated with the fluorescent probe 2,7 dichlorodihydro-fluorescein diacetate (H_2_DCFDA; 4 μmol/l in 1 ml; Molecular Probes) for 30 min at 37°C. After centrifugation (3000 g; 15 min; 4°C), protein content was quantified on the pellet. ROS were measured in 200 μl of supernatant using a Fluorescent Plate Reader (Perkin Elmer) at 535 nm under excitation at 490 nm. Fluorescence intensity was expressed as arbitrary units per milligram of protein.

### VMH S-nitrosylation measurement

The fourth day of the RH protocol, rats were injected with saline (S^3^S or I^3^S) and sacrificed 45 minutes later by decapitation. VMH were harvested, snap frozen and stored at −80°C. S-nitrosylation of soluble guanylyl cyclase (sGC) was determined using an S-nitrosylated protein detection kit (Cayman Chemical Company, Ann Arbor, MI) based on the method of Jaffrey *et al*. [22]. Briefly, the VMH were lysed in the dark and proteins were acetone precipitated. Free cysteine thiol groups were blocked according to the instructions in the kit. Existing nitrosylated thiol groups were then reduced to free thiols and labeled with biotin. sGC was immunoprecipitated using an antibody agaisnt the beta subunit of sGC (1∶10, Cayman Chemical Company) and a Western blot was performed against biotin (1∶75, S-nitrosylation detection kit, Cayman Chemical Company). Biotinylated thiols were visualized using horse radish peroxidase and quantified using Image J software against total protein content, which was quantified by Coomassie Blue staining. Only lanes which contained equal amounts of protein were used to determine the level of S-nitrosylation.

### CRR monitoring in response to acute insulin injection

Five to seven days before the beginning of the RH protocol, rats were anesthetized with sodium pentobarbital (50 mg/kg, IP, Ovation) and surgically implanted with vascular catheters in the right jugular vein. Catheters were filled with heparin (10 U/ml) and flushed every other day. Animals were allowed 5–7 days to recover from surgery before starting the RH protocol and were handled every day. Animals that did not recover to their pre-surgery body weights were excluded from the study. At the end of the RH protocol, following the fourth injection of saline or insulin, blood glucose was monitored every 15 minutes from −30 to 120 minutes post-insulin infusion *via* tail prick. Blood samples (500 µl) taken from the jugular catheter at 0, 60 and 120 minutes for subsequent measurement of plasma glucagon and epinephrine. For glucagon, 250 µl of blood was collected in chilled tubes containing EGTA (1.6 mg/ml, Sigma) and aprotinin (250 KIU, Sigma). For catecholamines, blood was collected in chilled tubes containing reduced glutathione (1.2 mg/ml, Sigma) and EDTA (1.8 mg/ml, Sigma).

### Hyperinsulinemic/hypoglycemic clamp

Rats were anesthetized with sodium pentobarbital (50 mg/kg, IP, Ovation) and surgically implanted with vascular catheters in the right jugular vein. Catheters were filled with heparin (10 U/ml) and flushed every other day. Additionally, rats were stereotaxically implanted with microinjection cannula guide in the 3^rd^ ventricle according to stereotaxic coordinates (from bregma: −2.0 mm anterior-posterior, 0 mm medial-lateral, and −8.0 mm dorsal-ventral). Animals were allowed 5–7 days to recover from surgery and were handled every day before performing the hypoglycemic clamp. Animals that did not recover to their pre-surgery body weights were excluded from the study. The day of the experiment, food was removed for six hours before the beginning of the clamp. Starting 60 minutes after ICV infusion (CSNO or artificial cerebrospinal fluid (aCSF) containing (in mM): 126 NaCl, 1.9 KCl, 1.2 KH_2_PO_4_, 26 NaHCO_3_, 2.4 CaCl_2_, 1.3 MgCl_2_, 300 mOsM, pH 7.4), rats were injected through the jugular catheter with an insulin bolus (rats: 0.4 U/kg) in order to decrease glycemia to ∼50 mg/dl within 30–40 minutes. This time course was used based on the results of Saberi *et al.* suggesting that brain *vs* peripheral glucose sensors predominate in CRR initiation when blood glucose decreases rapidly [23]. After this bolus, animals were perfused with insulin at 1.2 U/kg/h for 90 minutes. Glucose (20%) was co-perfused with insulin in order to maintain their plasma glucose level around 45–50 mg/dl. The concentration of blood glucose was measured every 10 minutes *via* tail prick. Blood samples (500 µl) taken from the jugular catheter were collected as described above at 0 and 90 minutes for subsequent measurement of plasma glucagon, epinephrine and norepinephrine. At the end of each experiment, cannula placement was verified by methyl-blue injection (Sigma).

### Plasma glucagon and catecholamines determination

Plasma glucagon concentrations were determined using commercially available radio-immunoassay kits (Linco Research). Plasma epinephrine and norepinephrine concentrations were analyzed by high-performance liquid chromatography using electrochemical detection (ESA, Acton).

### Measurement of membrane potential using fluorescence imaging plate reader membrane potential dye (FLIPR-MPD)

VMH dissociated neurons were prepared as previously described [13]. Neurons were visualized on an Olympus BX61 WI microscope with a ×10 objective equipped with a red filter for fluorometric imaging plate reader membrane potential dye (FLIPR-MPD) visualization (excitation, 548 nm; emission, 610–675 nm). Incubation of VMH neurons in extracellular solution (composition in mM: 25 HEPES, 121 NaCl, 4.7 KCl, 1.2 MgSO_4_, 5 NaHCO_3_, 2 CaCl_2_, 0.23 KH_2_PO_4_, 0.97 K_2_HPO_4_, and 2.5 glucose, pH 7.4) containing FLIPR-MPD (1 vial/667 mL extracellular solution) at 34°C began 30 min before and continued throughout the duration of all experiments. Images were acquired and analyzed with MetaMorph software (Universal Imaging) at 30-second intervals over the course of each experiment using a charge-coupled device camera (Cool Snap HQ; Photometrics) as extracellular glucose level was changed from 2.5 to 0.7 mM. The fluorescence intensity of each image (expressed as gray scale units per pixel) was normalized to that of the co-incubated fluorescent beads. Neurons which have a reversible increase in fluorescence of 12% or greater compared to that observed in 2.5 mM glucose are considered to be GI. An average percent change of >12% in FLIPR-MPD fluorescence intensity between 25 and 30 min [%ΔFLIPR-MPD (25–30)] defined a depolarized neuron. This threshold was defined as exceeding twice standard deviation of fluorescence variation observed with no glucose change (*i.e.* noise). Cell viability was confirmed at the end of each experiment by a 5-min exposure to 25 mM KCl. The percentage of depolarized neurons [%ΔFLIPR-MPD (25–30) >12%] in response to 2.5–0.7 mM glucose decrease per culture dish was recorded for each rat treatment groups.

### Data analysis

All data are presented as mean ± SEM. Statistical analysis was performed using Graphpad Prism 5.0 by two-way ANOVA or one-way ANOVA followed by Dunnett or Bonferoni post-hoc test or by paired or unpaired *t*-test as described in the figure legends. p<0.05 indicates statistical significance.

## Results

### Insulin-hypoglycemia increases the level of VMH ROS production

**Figure 1:**
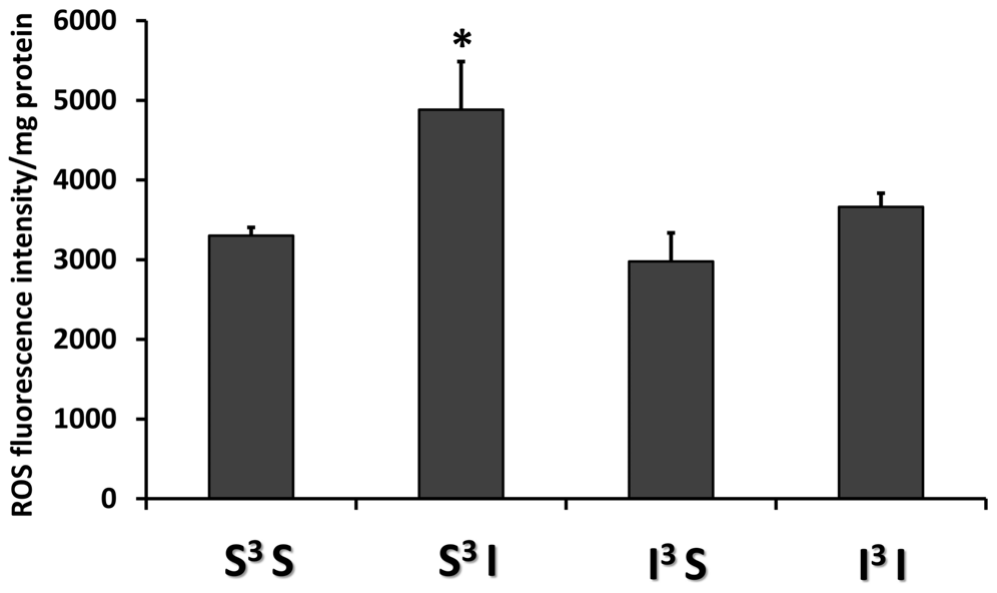
Hypoglycemia increases VMH ROS production. Quantification of fluorescence intensity of the ROS sensitive probe H_2_DCFDA in VMH of rats treated daily subcutaneously with saline or insulin (4 U/kg) for four consecutive days as presented in Table 1 (S^3^S: n = 16; S^3^I: n = 12; I^3^S: n = 4; I^3^I: n = 5). *: p<0.05 S^3^S *vs* S^3^I (One-way ANOVA followed by Bonferoni post-hoc test).

Singh *et al.* previously showed that insulin-induced hypoglycemia decreases the level of diencephalic antioxidant proteins catalase and glutathione (GSH) and increases the level of lipid peroxidation [17]. These data suggest that hypoglycemia increases hypothalamic ROS levels. In order to confirm these findings, we quantified the fluorescence intensity level of the ROS sensitive probe H_2_DCFDA (2′,7′-dichlorodihydrofluorescein diacetate) in the VMH of rats exposed to single or recurrent episodes of insulin-hypoglycemia. Rat treatment groups are represented in Table 1. As shown in Figure 1, the level of H_2_DCFDA fluorescence intensity was significantly increased by a factor of 1.5±0.6 in response to a single episode of insulin-hypoglycemia (Figure 1). Surprisingly, the basal VMH ROS level was not altered after recurrent episodes of insulin-hypoglycemia. Furthermore, a fourth episode of insulin-hypoglycemia failed to increase VMH ROS production (Figure 1). Together, these data are consistent with increased VMH ROS production after a single episode of hypoglycemia.

### RH increases the level of VMH S-nitrosylation

**Figure 2:**
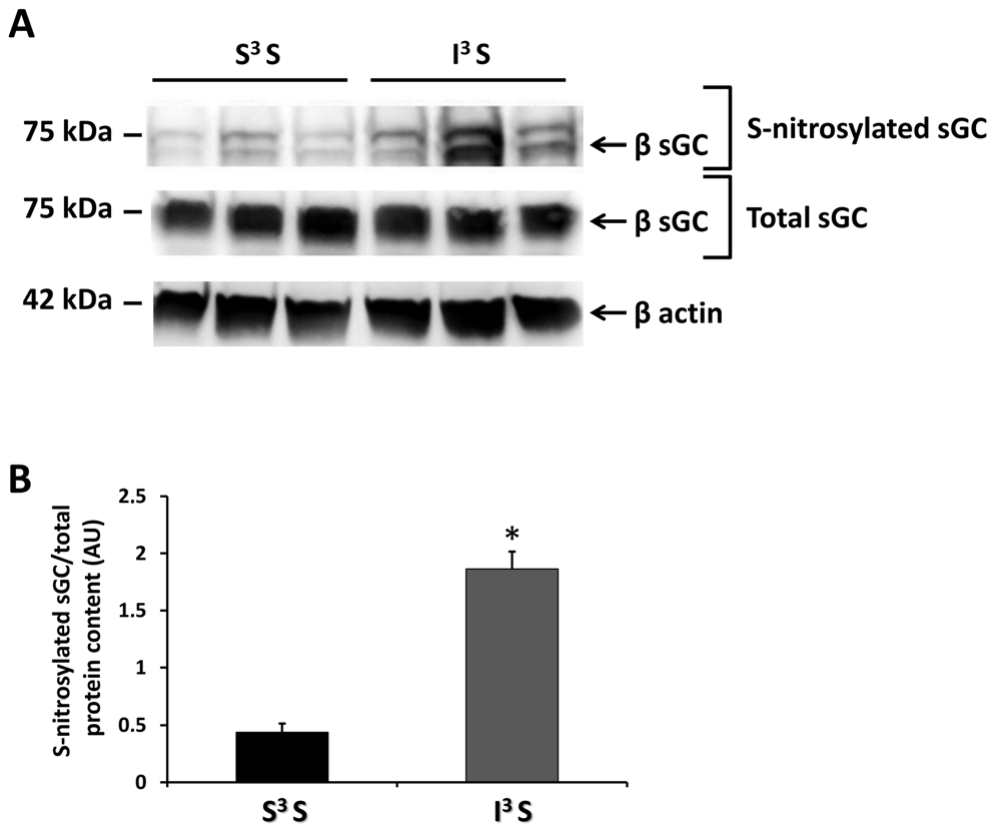
RH increases VMH S-nitrosylation. (A) Representative Western blot against VMH S-nitrosylated sGC (Upper panel), total sGC (Middle panel) and β actin (Bottom panel) from S3S and I^3^S rats. The lower band of the Upper panel represents the b-sGC subunit revealed using an anti-biotin switch-assay following pull down with anti-b-sGC. Since both a and b sGC subunits are known to be S-nitrosylated [16], the top band is probably the a subunit which would have been pulled down with the b subunit as an heterodimer with anti-b. (B) Relative S-nitrosylated sGC quantification normalized to total protein content. *: p<0.05 (n = 3; unpaired *t*-test).

We previously showed that insulin-hypoglycemia increases VMH NO production [12]. In the presence of elevated ROS levels, NO may interact with the reactive free-thiol cysteine in proteins to create a covalent modification, S-nitrosylation (SNO) [15]. The NO receptor sGC, which we have shown to be involved in CRR regulation, is a known target of S-nitrosylation [12], [16], [24]. To test our main hypothesis that the level of S-nitrosylation is increased after RH, we quantified the level of S-nitrosylated sGC in the VMH. Our data show that RH increased the basal level of S-nitrosylated sGC by a factor of 4.3±0.34 (n = 3; Figure 2A, B) where total VMH sGC level was not affected by RH (S^3^S: 1.04±0.04 *vs* I^3^S: 1.046±0.06 AU total sGC/β actin; n = 3; p >0.05, unpaired *t*-test; Figure 2A).

### Increased VMH S-nitrosylation impairs the CRR

**Figure 3:**
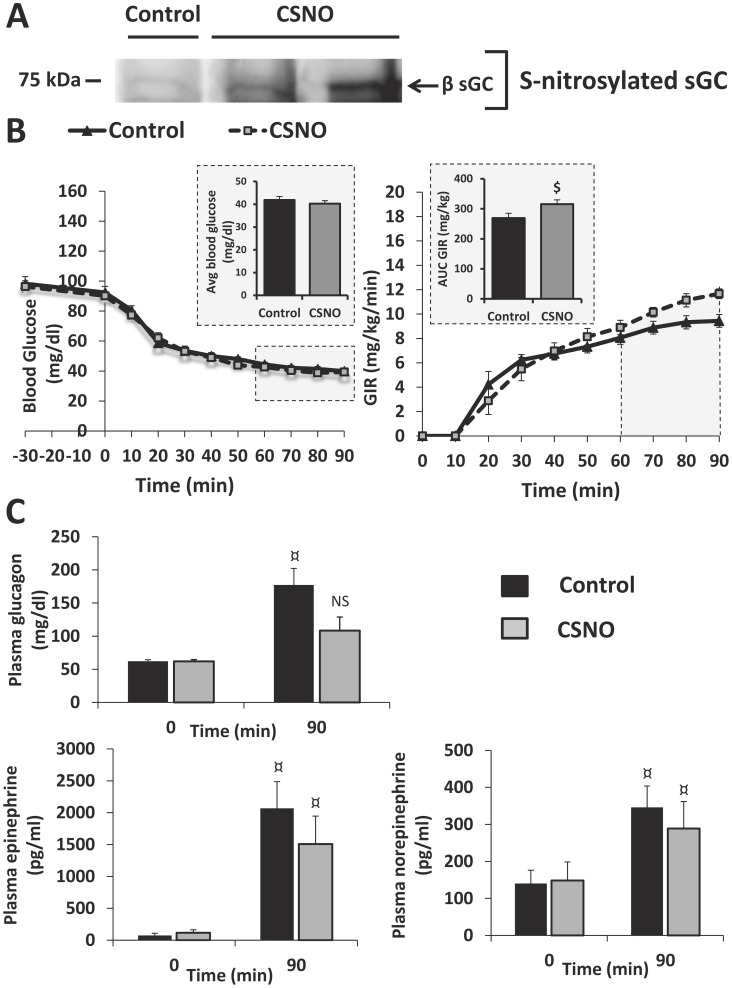
ICV CSNO injection increases VMH sGC S-nitrosylation and impairs the CRR. (A) Representative Western blot against VMH S-nitrosylated sGC from control and CSNO treated rats. (B–C) Blood glucose level (B left panel; n = 9; *Inset*: average blood glucose during the last 30 minutes of the clamp); glucose infusion rate (GIR; B right panel; n = 9; *Inset:* AUC of the GIR during the last 30 minutes of the clamp); plasma glucagon (C upper left panel; n = 5), epinephrine (C lower left; n = 5) and norepinephrine levels (C lower right panel; n = 5) during hyperinsulinemic/hypoglycemic clamp (1.2 U/kg/h) of animals injected ICV with aCSF (Control) or CSNO (0.5 mM). *: p<0.05 *vs* controls (Two-way ANOVA followed by Bonferoni post-hoc test); $: p<0.05 *vs* control (unpaired *t*-test); ¤: p<0.05 time 0 *vs* time 90 (paired *t*-test); NS: p>0.05 time 0 *vs* time 90 (paired *t*-test).

We hypothesized that increased VMH S-nitrosylation is involved in CRR impairment after RH. To determine whether increased VMH S-nitrosylation is associated with an impaired CRR, we injected rats with the NO donor and nitrosylating agent S-nitroso-L-cysteine (CSNO; 0.5 mM) intracerebroventricularly (ICV) in the third ventricle and analyzed the CRR using a hyperinsulinemic/hypoglycemic clamp. First, as expected, ICV CSNO injection increased the level of VMH S-nitrosylated sGC (Figure 3A). Following ICV CSNO injection, the glucose infusion rate (GIR) necessary to maintain the blood glucose level around 45 mg/dl was significantly increased during the last 30 minutes of the clamp (Figure 3B). This increase in the GIR was associated with reduced glucagon secretion. In control animals, 90 min after the beginning of the clamp, plasma glucagon, epinephrine and norepinephrine levels were significantly increased (Figure 3C). CSNO injection did not alter plasma epinephrine and norepinephrine release. However, in CSNO treated rats, plasma glucagon level was not significantly increased during the hyperinsulinemic/hypoglycemic clamp, unlike control (Figure 3C). These data suggest that increased VMH S-nitrosylation is associated with CRR impairment.

### NAC pre-treatment prevents VMH S-nitrosylation and impaired CRR after RH

**Figure 4:**
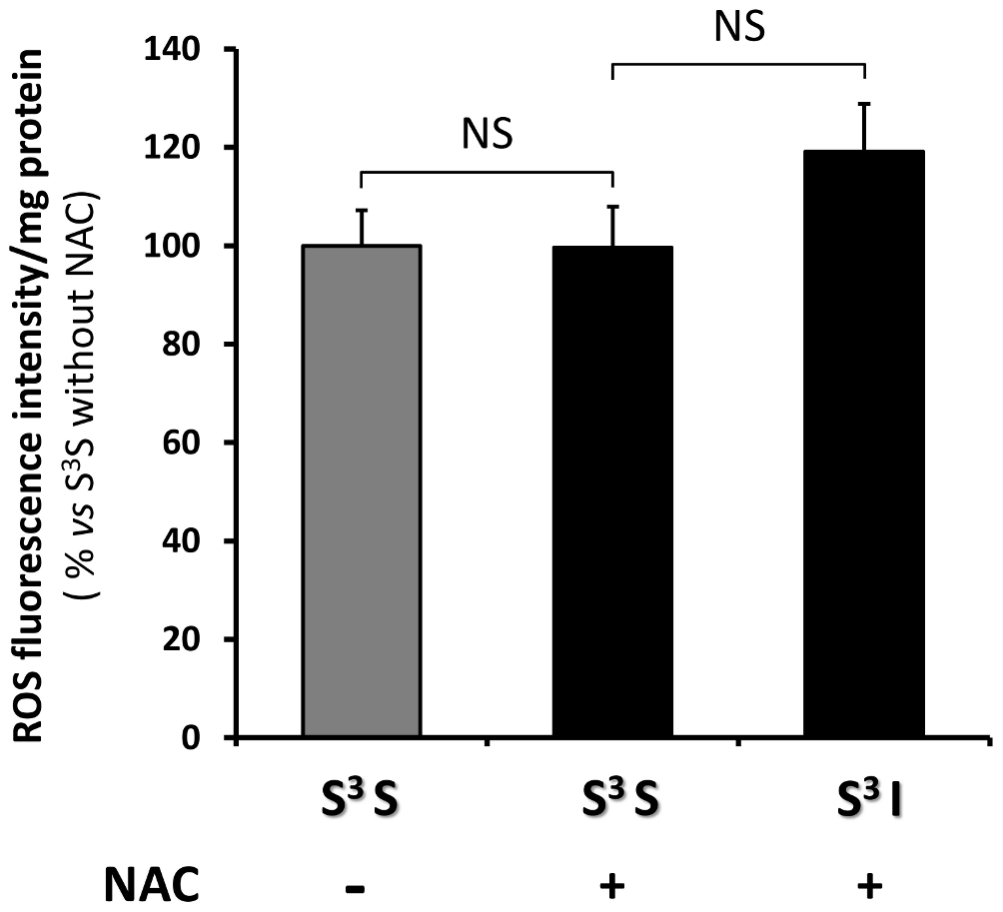
NAC treatment prevents hypoglycemia-induced increased VMH ROS production. Quantification of VMH fluorescence intensity of the ROS sensitive probe H_2_DCFDA of rats injected SC with saline or insulin (4 U/kg) and treated or not with NAC (5 g/l) in their drinking water. Data are expressed as % of control rats not treated with NAC and injected with saline (S^3^S). n number: S^3^S: n = 16; S^3^S + NAC: n = 8; S^3^I + NAC: n = 8. NS: p>0.05 (One-way ANOVA).

**Figure 5:**
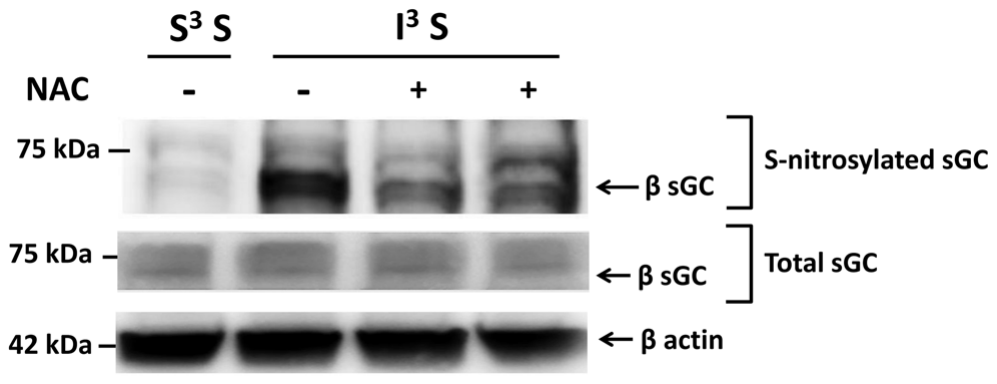
NAC prevents increased VMH S-nitrosylation after RH. Representative western blot against VMH S-nitrosylated sGC (Top) or total sGC (Bottom) from control (S^3^S) and RH rats (I^3^S) pre-treated or not with NAC. The lower band of the Upper panel represents the β-sGC subunit revealed using an anti-biotin switch-assay following pull down with anti- β-sGC. The top band is probably the α subunit which would have been pulled down with the β subunit as an heterodimer with anti-β.

**Figure 6:**
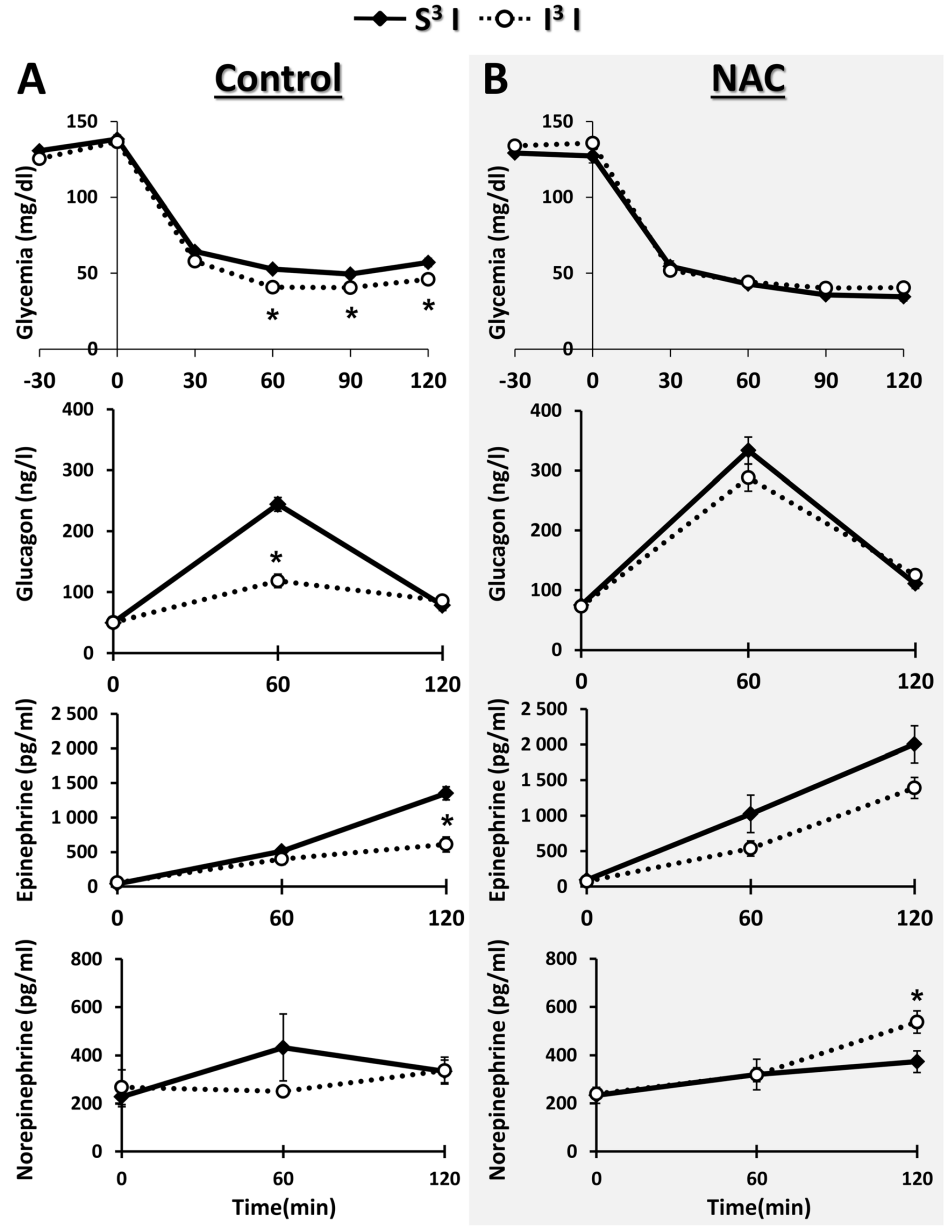
NAC prevents the impaired CRR following RH. Blood glucose levels (first top panel), plasma glucagon (second panel), epinephrine (third panel) and norepinephrine levels (lower panel) in response to insulin-hypoglycemia in control (A) and NAC pre-treated (B) rats following 3 consecutive daily injections of either insulin (S^3^I) or saline (I^3^I). *: p<0.05 S^3^I *vs* I^3^I (Repeated measures Two-way ANOVA followed by Bonferoni post-hoc test within control and NAC pre-treated rats).

We hypothesized that RH-induced VMH sGC S-nitrosylation and CRR impairment is the consequence of concomitant ROS and NO production. Thus, prevention of ROS production during hypoglycemia should attenuate or prevent S-nitrosylation and the impaired CRR. To test this hypothesis, we treated rats with N-acetyl-cysteine (NAC) in their drinking water (0.5%) for 9 days prior to and during RH and analyzed the level of VMH S-nitrosylation and the CRR. NAC treatment did not affect basal VMH ROS level (Figure 4). However, while insulin-hypoglycemia increased VMH ROS production by 50% in control rats (Figure 1), insulin-hypoglycemia did not significantly increase VMH ROS levels in NAC treated rats (Figure 4). Furthermore, NAC pre-treatment significantly decreased the level of VMH sGC S-nitrosylation following RH (I^3^S: 1.86±0.15 *vs* I^3^S + NAC: 0.69±0.15 AU/total protein content; n = 3; unpaired *t*-test; p<0.05; Figure 5) without affecting total sGC level. Finally, as shown in Figure 6, NAC pre-treatment prevented CRR impairment following RH. In control animals, blood glucose levels of rats exposed to RH fell further and glucagon and epinephrine production were blunted in response to a fourth episode of insulin-hypoglycemia (I^3^I) as compared to saline-injected rats exposed to one episode of hypoglycemia (S^3^I). A trend toward decreased plasma norepinephrine levels was also observed after RH in I^3^I animals (Figure 6A). However, following NAC pre-treatment there were no significant differences between the blood glucose decline or glucagon and epinephrine production in rats exposed to a single (S^3^I) or recurrent (I^3^I) episodes of insulin-hypoglycemia. In addition, plasma norepineprhine levels were increased after RH in I^3^I animals treated with NAC (Figure 6B). Thus, NAC prevents RH-induced VMH S-nitrosylation and CRR impairment.

### NAC pre-treatment prevents impaired VMH GI neuronal response to decreased glucose after RH

**Figure 7:**
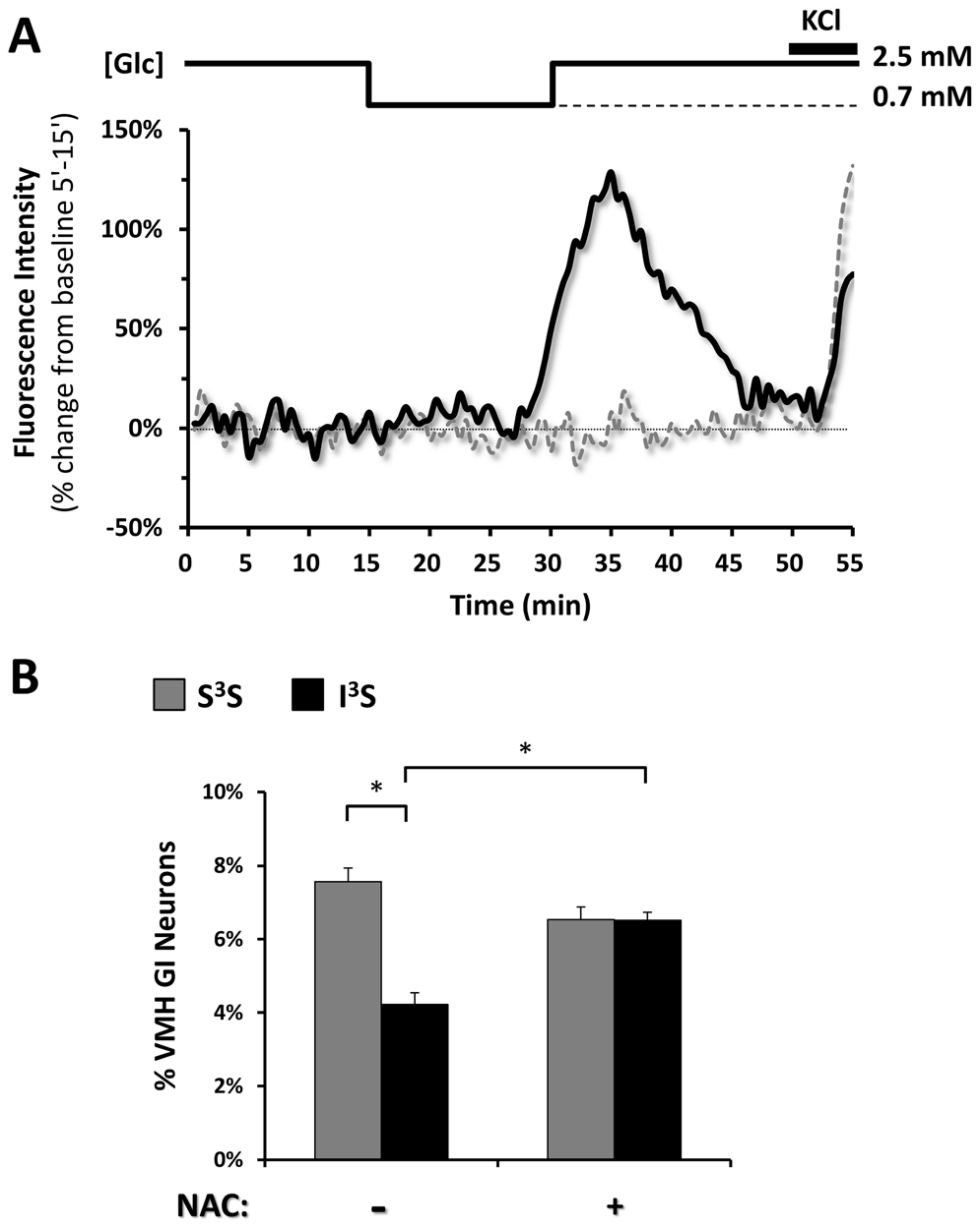
NAC prevents VMH GI neuron activation by decreased glucose. (A) Representative fluorescence intensity measurement from FLIPR-MD recording of a VMH GI (black full line) and a non-glucose sensitive neuron (gray dotted line). The black dotted line represents the fluorescence intensity baseline. Glucose concentration changes and KCl application are schematically displayed above each recording. (B) Percentage of VMH GI neurons from S^3^S or I^3^S rats pre-treated or not with NAC which depolarized in response to a 2.5–0.7 mM glucose decrease quantified using changes in FLIPR-MPD fluorescence intensity. *: p<0.05 (Two-way ANOVA; group differences were determined by One-Way ANOVA followed by Bonferoni post-hoc test).

We previously showed that the glucose response of VMH GI neurons to decreased glucose is impaired after RH [11]. We confirm these data using membrane potential sensitive imaging in cultured VMH neurons which quantifies the number of VMH GI neurons observed in response to a 2.5–0.7 mM glucose decrease (Figure 7). The number of VMH GI neurons was significantly decreased by 37.5±7.78% after RH. Interestingly, while NAC pre-treatment did not alter the number of VMH GI neurons in naïve rats, NAC prevented the decreased number of VMH GI neurons after RH (Figure 7B).

## Discussion

We hypothesized in the present study that increased VMH S-nitrosylation of key glucose sensing proteins such as sGC after RH may impair the CRR to subsequent hypoglycemia. Our data show that VMH sGC S-nitrosylation is indeed increased after RH where the CRR is impaired. Interestingly, we found that ICV CSNO increases VMH sGC S-nitrosylation and also impairs the CRR. Finally, pre-treating rats with the antioxidant NAC prevents increased VMH sGC S-nitrosylation, impaired glucose sensing by GI neurons and the blunted CRR following RH. Thus, our study supports the hypothesis that preventing increased VMH sGC S-nitrosylation may prevent glucose sensing and CRR impairment following RH.

We previously showed that hypoglycemia-induced VMH NO production plays a critical role in the CRR through a sGC dependent mechanism [12]. In addition to its sGC-dependent signaling pathway, NO affects other signaling pathways through S-nitrosylation. S-nitrosylation is a non-enzymatic reaction consisting of the covalent attachment of a NO moiety to the thiol side chain of cysteine to form SNO adducts, altering the properties of proteins/enzymes [15], [25]. It is noteworthy that the NO bond is labile which makes S-nitrosylation difficult to detect and quantify. Nevertheless, over the past decade, the number of reported substrates for S-nitrosylation has grown significantly [22]. This is consistent with the ubiquity of reactive-site thiols across protein classes. sGC is one of the many target proteins of S-nitrosylation [16]. We found herein that VMH sGC S-nitrosylation is increased after RH, a condition where the CRR is impaired. The fact that the NO donor and nitrosylating agent CSNO increases VMH sGC S-nitrosylation and impairs the CRR further support the idea that increased VMH S-nitrosylation plays a critical role in CRR impairment. However, CSNO may also interact with side chain of tyrosine to create tyrosine nitration [26]. Thus, we cannot exclude that tyrosine nitration might also be involved in CSNO-induced CRR impairment and more generally in the development of HAAF.

The current findings support our hypothesis that VMH GI neurons control the CRR through a NO-sGC dependent pathway [11], [12], [13]. We have found that the response of VMH GI neurons to decreased glucose is impaired in a number of conditions where the CRR is impaired [11], [12], [27], [28]. For example, electrophysiological evaluation of VMH GI neurons in brain slices from rats after RH showed that the threshold to activate VMH GI neurons shifts to lower glucose levels. We now extend these results by evaluating the glucose sensitivity of isolated VMH GI neurons from rats after RH with and without NAC treatment using the FLIPR-MPD imaging technique. The FLIPR-MPD technique is not sensitive enough to quantify the absolute degree of depolarization in response to a specific glucose decrease. Rather, we are only able to detect the percentage of VMH neurons which depolarize over a noise threshold. In the present study, fewer VMH neurons from RH treated rats depolarized in response to decreased glucose. Thus, we interpret the present results to mean that fewer VMH GI neurons depolarized over the FLIPR-MPD depolarization threshold as glucose decreased rather than an actual decrease in the number of VMH GI neurons. Importantly, NAC prevents RH-induced impairment of both the CRR and glucose sensing by VMH GI neurons. These data provide further support for a role of VMH GI neurons in regulating the CRR.

The mechanism underlying RH-induced increased VMH S-nitrosylation remains a question of interest. Increased NO production via the inducible NOS (iNOS) has been shown to increase S-nitrosylation levels [29]. However, we previously showed that iNOS activity is not altered in response to either acute or recurrent hypoglycemia [12], [30]. S-nitrosylation may also occur when NO is produced under oxidative conditions where NO and ROS can interact [31]. In support of this hypothesis, we found that hypoglycemia increases both VMH ROS and NO levels [30]. Increased VMH ROS level during hypoglycemia is consistent with a previous study showing that hypoglycemia decreased expression of antioxidant factors and increased lipid peroxidation [17]. To further support the hypothesis that VMH ROS production is involved in hypoglycemia-induced increased S-nitrosylation levels, we found that treating rats with the antioxidant NAC prevents both increased VMH ROS and S-nitrosylation levels. NAC, as a precursor for glutathione biosynthesis is a very powerful antioxidant that also reacts directly with electrophiles [19]. NAC is used *in vitro* and *in vivo* to decrease ROS and S-nitrosylation levels [16], [24], [32]. In human or animal models NAC is used to prevent and reverse negative clinical outcomes such as nitrate tolerance and paracetamol overdose [33], [34]. Surprisingly, NAC treatment did not decrease basal VMH ROS level as one might have expected. It is possible that while NAC treatment at the dose of 5 g/l (∼0.55 g/kg/day) was not sufficient to alter basal cell redox status, this dose was able to prevent any small increase in ROS production. This idea is consistent with the study from Kamboj *et al.* showing that NAC given at 1.5 g/kg/day in the drinking water did not alter basal glutathione or lipid peroxidation levels in control rats [19]. In addition, our data suggest that VMH ROS production does not play a role in CRR initiation under basal conditions. That is, while NAC pre-treatment inhibited hypoglycemia-induced VMH ROS production, it did not affect CRR hormones secretion in response to a single episode of hypoglycemia. In contrast, blood glucose level did fall further in response to insulin-hypoglycemia in NAC-treated rats. This is consistent with Song *et al.*'s study showing that NAC increases insulin sensitivity in an animal model of T2DM [35]. Thus, the lower glucose nadir observed in non-RH NAC treated rats is likely due to an increased sensitivity to insulin injection.

The next point which needs to be addressed is the mechanism involved in hypoglycemia-induced VMH ROS production. VMH ROS formation may be consequent to increased NO production. In support of this, NO inhibits mitochondrial cytochrome c and blocks mitochondrial respiration which consequently increases superoxide anion O_2_^•−^ production [36]. Increased NO production during hypoglycemia also activates the neuronal NAPDH oxidase which could, in turn, increase ROS through a mitochondria-independent pathway [37]. On the other hand, the amount of NO produced by nNOS is relatively low when compared to that produced by iNOS. This suggests that these NO-dependent mechanisms may not be the only cause of increased ROS levels. In support of this, lipid peroxidation, an indirect marker of ROS production, is increased in response to insulin-hypoglycemia in the cortex [17]. However, we did not observe increased cortical NO release following insulin-hypoglycemia [12]. Decreased antioxidant defenses may also explain increased ROS levels. Hypoglycemia is associated with decreased activity of the antioxidant enzymes catalase and super oxide dismutase [17]. In addition, insulin is also known to increase VMH ROS production [38]. Thus, insulin injection used in our clinically relevant model of hypoglycemia may also contribute to the increased VMH ROS production. Further studies are clearly needed to determine which mechanism(s) underlie insulin-hypoglycemia-induced ROS production.

Furthermore, we do not yet understand why increased VMH S-nitrosylation persists after RH when neither VMH NO nor ROS levels remain elevated. Recently, it was suggested that protein S-nitrosylation is more stable than initially thought, and is mainly dependent upon a dynamic denitrosylation process such as that regulated by the thioredoxin system [39], [40]. As such, we can infer that, once induced, protein S-nitrosylation lasts for a certain period of time in the absence of nitrosylating agents (NO, ROS) and/or if thioredoxin reducing ability is impaired. Thus, following a transitory burst of ROS and NO production in response to a single episode of insulin-hypoglycemia, VMH S-nitrosylation level remains increased for some time. It is well established that the CRR remains impaired for approximately 2 weeks following RH, after which a normal CRR is restored if there have been no further hypoglycemic episodes during that time [3]. We speculate that the persistent increase in VMH S-nitrosylation could play a role in the development of the sustained CRR impairment after RH at least in the initial phase. It is possible that elevated protein nitrosylation and/or reduced NO signaling leads to downstream changes in other intracellular signaling pathways which explain the duration of HAAF. However, further studies of the correlation between the duration of HAAF, VMH S-nitrosylation and the signaling pathways involved are needed to support or refute this hypothesis. In addition, the question whether RH-induced increased S-nitrosylation is specific to the VMH remains. Increased S-nitrosylation may happen in brain areas not known to be involved in the regulation of the CRR such as the cortex. However, even though ROS production is increased in the cortex in response to hypoglycemia, we previously showed that NO production is not [12], [17]. Further studies are still needed to determine whether S-nitrosylation is increased in extra-hypothalamic regions after RH.

The most important finding of our study is that NAC pre-treatment prevents both increased VMH S-nitrosylation, decreased number of VMH GI neurons and blunted glucagon and epinephrine secretion in response to RH. While the present study does not establish a direct causal link between increased VMH S-nitrosylation and CRR impairment, when considered in the context of our previous work, our data strengthen our hypothesis that VMH S-nitrosylation plays an important role in the impaired CRR following RH. That is, we have shown that inhibiting the NO-sGC pathway specifically in the VMH impairs the CRR [12]. Since S-nitrosylation of sGC causes NO resistance, VMH S-nitrosylation would certainly impair NO-sGC signaling and as a result impair the CRR. However, since NAC was administered orally, we cannot exclude VMH S-nitrosylation independent effects in the prevention of CRR impairment. Nevertheless, our study suggests that NAC might be an important clinical therapy for the treatment of HAAF. The dose of NAC used in drinking water (0.5%) corresponds approximately to 0.55 g/kg/day based on the rats' water intake. This dose of NAC is equivalent to that used clinically in humans to reverse the liver damage following paracetamol intoxication [18]. This is particularly relevant since NAC is approved for use in humans in the US as well as in Europe. As mentioned above, the insulin dose for patients with T1 or T2DM using insulin therapy would have to be carefully monitored when initiating NAC therapy since NAC increases insulin sensitivity [35]. This is especially true since our study only evaluated NAC as a prophylactic therapy given before hypoglycemia occurred. Additional studies need to be performed to determine whether NAC can be used as a treatment to reverse HAAF following RH. However, since improving insulin sensitivity would allow patients to reduce their insulin doses, prophylactic NAC therapy may be very efficacious for patients with T1 or T2DM both in terms of preventing HAAF and improving overall health.

In conclusion, our study shows that increased VMH ROS production may contribute to the development of HAAF following RH. Moreover, the mechanism by which ROS exerts its deleterious effects on the CRR may involve S-nitrosylation of key proteins involved in hypothalamic glucose sensing (e.g., sGC). This study also shows that preventing S-nitrosylation with NAC may be useful for patients with T1 or advanced T2DM treated with insulin therapy. Since NAC is already FDA approved for use in humans, our study highlights an exciting new therapy which could be rapidly adapted to treat HAAF in patients with T1 or advanced T2DM.
